# Impact of beta-blocker therapy on thoracic aorta 3D wall shear stress in patients with bicuspid aortic valve

**DOI:** 10.1186/1532-429X-16-S1-O47

**Published:** 2014-01-16

**Authors:** Bradley D Allen, Pim van Ooij, Alex J Barker, Jeremy D Collins, James C Carr, Michael Markl, Preeti Kansal

**Affiliations:** 1 Radiology, Northwestern University, Chicago, Illinois, USA; 2 Biomedical Engineering, Norhtwestern University, Chicago, Illinois, USA; 3 Medicine - Cardiology, Norhtwestern University, Chicago, Illinois, USA

## Background

Beta-blockers are the recommended medical treatment for slowing ascending aorta (AAo) dilation in patients with bicuspid aortic valve (BAV). Wall shear stress (WSS) has been shown to promote endothelial cell dysfunction [1] and AAo WSS may play a role in aortic aneurysm growth. Time-resolved 3D phase contrast (4D flow) MRI allows for the quantification of 3D WSS regionally in the thoracic aorta. The aim of this study was to assess changes in thoracic aorta WSS associated with β-blocker therapy in BAV patients.

## Methods

BAV patients on β-blockers (BB+) (n = 10, M:F = 8:2, age: 53 ± 11 years) or not on β-blockers (BB-) (n = 10, M:F = 9:1, age: 51 ± 15 years) underwent 4D flow MRI as part of this IRB-approved study. Groups were matched by BAV morphology (all right-left fusion), systolic blood pressure (BB+: 137 ± 12 mmHg, BB -: 132 ± 17 mmHg, p = 0.48), degree of aortic stenosis, and AAo diameter (BB+: 4.1 ± 0.7 cm, BB-: 3.6 ± 0.4 cm, p = 0.07). Five patients in each group were concurrently treated with ACE-inhibitors or angiotensin receptor blockers. Data analysis included correction for eddy currents and velocity aliasing and 3D segmentation of the thoracic aorta (MIMICS, Materlise, Belgium). Peak systolic WSS (WSS_sys_) was calculated within the vessel using the method described by van Ooij[2]. The aorta was divided into ascending, arch, and descending regions, and max and mean WSS_sys _were calculated in each region. WSS_sys_maximum intensity projections (MIP) were mapped onto a sagittal view of each aorta for visual comparison. Quantitative results were compared using Student's t-test. Spearman (r_S_) or Pearson (r) correlation was performed as appropriate.

## Results

**Figure 1:**
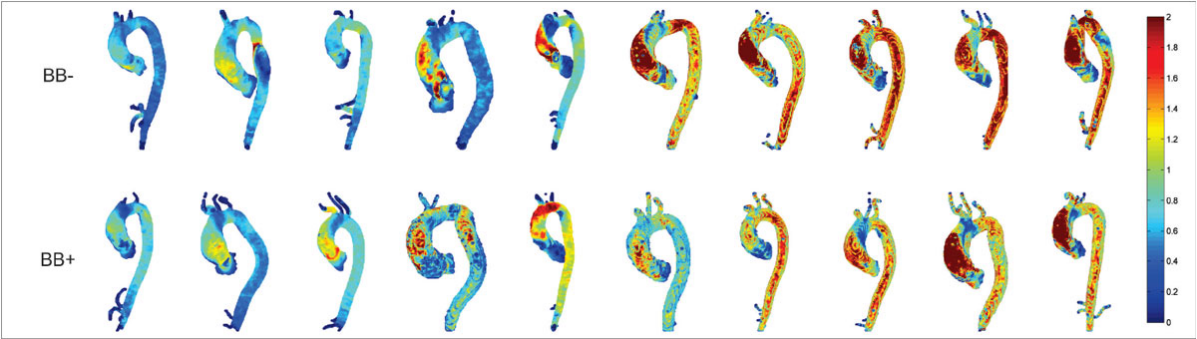
**Peak Systolic Wall Shear Stress (WSS_sys_) maximum intensity projections in the study cohort**. Values for WSS_sys _are in N/m^2^. Note that there is a large variation across both groups in terms of WSS_sys _values and where the maximum WSS is located. In general, patients appear to have WSS_sys _angled toward the lateral wall of the ascending aorta, however, there appears to be fewer patients with this eccentric WSS distribution in the β-blocker group (BB+).

No statistical difference in max or mean WSS_sys _was observed between BB+ and BB- groups at any region along the aorta, although values were consistently lower in the BB+ group (Table 1). Max WSS_sys _in the AAo showed no correlation with aortic diameter (r = .289, p = 0.22) but did correlate with the degree of aortic stenosis (r_S _= 0.44, p = 0.05). WSS_sys _MIPs for all subjects are shown in Figure 1. The magnitude and regional distribution of WSS_sys_was highly variable between individuals for both groups. Decreased eccentricity of WSS_sys _in the AAo was observed in BB+ patients.

**Table 1:** Max and mean peak systolic wall shear stress (WSS_sys_) regionally along the aorta in bicuspid aortic valve patients being treated with beta-blockers (BB+) or not receiving beta-blockers (BB-).

	Max WSS (N/m^2^)			Mean WSS (N/m^2^)		
	BB+	BB-	p-value	BB+	BB-	p-value
Ascending Aorta	2.9 ± 1.7	3.2 ± 1.8	0.70	0.9 ± 0.3	1.0 ± 0.4	0.40
Arch	1.7 ± 0.5	2.3 ± 1.2	0.20	0.9 ± 0.3	1.2 ± 0.5	0.15
Descending Aorta	2.1 ± 0.8	2.4 ± 1.1	0.57	1.3 ± 0.4	1.4 ± 0.1	0.66

## Conclusions

Our results suggest that the impact of β-blocker therapy on the degree of WSS in the thoracic aorta is limited, but treatment may alter AAo WSS distribution. The high inter-individual variability of 3D WSS highlights the potential diagnostic value of 4D flow MRI WSS quantification for individualized assessment β-blocker effectiveness in BAV aortopathy. A prospective study in a large number of patients pre- and post-treatment is required to better isolate the impact of β-blockers in this population.

## Funding

NIH NCI 5R25CA132822-04, NIH NHLBI R01HL115828; AHA13SDG14360004, BAV Program at the Bluhm Cardiovascular Institute.
